# Stress Resistance Screen in a Human Primary Cell Line Identifies Small Molecules That Affect Aging Pathways and Extend *Caenorhabditis elegans*’ Lifespan

**DOI:** 10.1534/g3.119.400618

**Published:** 2019-12-26

**Authors:** Peichuan Zhang, Yuying Zhai, James Cregg, Kenny Kean-Hooi Ang, Michelle Arkin, Cynthia Kenyon

**Affiliations:** * Department of Biochemistry and Biophysics,; ‡ Department of Pharmaceutical Chemistry, Small Molecule Discovery Center, University of California, San Francisco, CA, 94143 and; † Calico Life Sciences LLC, South San Francisco, CA, 94080

**Keywords:** Small molecule screen, oxidative stress, stress resistance, NRF2, aging, *C. elegans*, WI-38

## Abstract

Increased resistance to environmental stress at the cellular level is correlated with the longevity of long-lived mutants and wild-animal species. Moreover, in experimental organisms, screens for increased stress resistance have yielded mutants that are long-lived. To find entry points for small molecules that might extend healthy longevity in humans, we screened ∼100,000 small molecules in a human primary-fibroblast cell line and identified a set that increased oxidative-stress resistance. Some of the hits fell into structurally related chemical groups, suggesting that they may act on common targets. Two small molecules increased *C. elegans*’ stress resistance, and at least 9 extended their lifespan by ∼10–50%. We further evaluated a chalcone that produced relatively large effects on lifespan and were able to implicate the activity of two, stress-response regulators, *NRF2*/*skn-1* and *SESN*/*sesn-1*, in its mechanism of action. Our findings suggest that screening for increased stress resistance in human cells can enrich for compounds with promising pro-longevity effects. Further characterization of these compounds may reveal new ways to extend healthy human lifespan.

In animals, mutations in nutrient, energy and stress-sensing genes, such as IGF-1-axis and mTOR-signaling genes, extend youthfulness and lifespan and counter age-related disease (Fontana *et al.* 2010; Kenyon 2010; Bartke 2011). These genes are members of large networks with multiple components, some of which could potentially serve as targets for pharmacological interventions to increase healthy lifespan. In fact, consistent with genetic perturbations, small molecules that inhibit mTOR (rapamycin) or activate AMP kinase (metformin) have been reported to prolong lifespan in several different species (Ding *et al.* 2017), albeit modestly in the case of metformin in mice (Martin-Montalvo *et al.* 2013). Some of these small molecules have been used in humans to treat diabetes and cancer, two diseases that afflict the aging population. Together these findings suggest that interventions targeting components of this network could extend the healthy life of humans.

In addition to targeting human orthologs of proteins known to influence longevity in animals, an alternative approach might be to identify promising molecules in phenotypic screens for cellular correlates of animal longevity. One such correlate is resistance to environmental stress. Many long-lived mutants and their cells are resistant to multiple types of stressors (Miller 2009). In addition, cells from long-lived wild animals, such as brown bats and naked mole-rats, are resistant to oxidizing radicals, heavy metals and DNA-damaging agents (Lewis *et al.* 2012; Salmon *et al.* 2008; Harper *et al.* 2007). Third, forkhead box O (FOXO) proteins (such as FOXO3) and nuclear factor, erythroid 2 like 2 (NFE2L2, aka NRF2), key regulators of stress responses, can promote longevity in several species, including worms (Kenyon *et al.* 1993; Libina *et al.* 2003; Ogg *et al.* 1997; Tullet *et al.* 2008; Bishop and Guarente 2007), flies (Giannakou *et al.* 2004; Hwangbo *et al.* 2004; Sykiotis and Bohmann 2008), and likely mice (Steinbaugh *et al.* 2012; Leiser and Miller 2010; Shimokawa *et al.* 2015). In humans, exceptional longevity-associated *FOXO3A* polymorphisms have been identified in multiple cohorts (Kenyon 2010; Joshi *et al.* 2017; Kolovou *et al.* 2017), and NRF2 activation and mTOR inhibition have been shown to delay senescence in human fibroblasts (Kapeta *et al.* 2010; Walters *et al.* 2016). As proof of concept, genetic screens for organismal stress resistance in yeast, worms and plants have enriched for mutants that live long (de Castro *et al.* 2004; Kim and Sun 2007; Kennedy *et al.* 1995; Cao *et al.* 2003). Conversely, by screening a library of compounds with known mammalian pharmacological targets, Ye and co-workers identified 60 that promoted longevity of *C. elegans*, and of these, 33 increased worms’ resistance to oxidative stress (Ye *et al.* 2014). Several lines of evidence indicate that increased oxidative-stress resistance may not be the cause of extended longevity (Slack *et al.* 2011; Pérez *et al.* 2009). However, these findings show that selection strategies for stress resistance can be used to enrich for longevity regulators.

To this end, we carried out a high-throughput screen for small molecules that enhance resistance to hydrogen peroxide-induced oxidative stress in a human primary cell line. We further characterized our hit molecules in assays related to known longevity pathways and found that certain molecules activated targets of NRF2/SKN-1. We also found that some molecules could extend the lifespan of *C. elegans*.

## Materials and Methods

### Small molecule screen library

We screened a “diversity library” that contains 104,121 small molecules (∼24K from ChemBridge; ∼50K from ChemDiv; and ∼30K from SPECS) selected to maximize the coverage of chemical space. Compounds were provided as 10 mM stocks by the Small Molecule Discovery Center (SMDC), and chemical information, including structures, simplified molecular-input line-entry system (SMILES) IDs, PubChem links and possible *in silico* docking, is available for query. The SMILES of 209 primary screen hits are shown in Supplemental Table 1.

### Cell culture & H_2_O_2_-resistance screen

For the screen, we used human lung-derived WI-38 cells at mid population doubling levels (PDLs ∼30; these cells undergo replicative senescence at PDL ∼50). Briefly, WI-38 cells were cultured in OptiMEM (Life Technologies) supplemented with 10% fetal bovine serum (FBS). In a 384 multi-well format, 2,000 cells were added in 50 μl medium per well, using a WellMate (Thermo Fisher) liquid dispenser and cultured in a CO_2_ incubator at 37° for 24 hr. Small molecules were introduced from stock plates with a 384-well formatted set of 50 nL stainless steel pin-tool (V&P Scientific) on a Biomek FXP (Beckman) automation workstation to 10 μM final concentration (in 0.1% DMSO), a typical single-concentration assay dose shown to produce high structural diversity in many small-molecule screens (Walters and Namchuk 2003). The negative (0.1% DMSO-treated cells, n = 32) or positive (50 nM calyculin-treated cells, n = 32) controls were assigned to columns 1 & 2 or 23 & 24 respectively on the 384-well cell-culture plates to reduce potential cross-contamination with library compounds. Twenty-four hours later, the cells were subjected to a 3-hour stress treatment with 700 μM H_2_O_2_, and viability was assessed using CellTiter-Glo reagent (Promega), measuring luminescence signals on an Analyst HT (Molecule Devices) plate reader.

### Imaging analysis

A high-content imaging assay was developed as a secondary approach to validate our screen hits. Briefly, cells were pre-incubated with small molecules for 24 hr and then subjected to H_2_O_2_ for 3 hr (in several experiments; multiple, consecutive times points were included for analysis). Hoechst 33342 (Life Technologies) (10 μg/ml final concentration) and propidium iodide (Life Technologies) (2.5 μM final concentration) were added for the last 30 min. Fluorescent images of cells were collected on the INCell Analyzer 2000 automated microscope (GE Healthcare) (10X objective) and further analyzed with the Developer ToolBox software (GE Healthcare; version 1.9).

### RNA-seq analysis

For RNA-seq analysis, WI-38 cells were treated with Gr-4D (2.5 μM final, a dose confirmed to promote H_2_O_2_-resistance in a paralleled experiment – see Supplemental Figure 6) from two different vendors (CB, ChemBridge; MP, MolPort), 0.1% DMSO control or no DMSO mock control (n = 6 each) in the absence of H_2_O_2_ for 24 hr and then processed for RNA isolation with the RNeasy kit (Qiagen). Total RNA (375ng, normalized) was used for each sample to prepare Illumina TruSeq Stranded mRNA library, following the manufacturer’s instructions. Multiplexed samples were analyzed on an Illumina HiSeq 4000 to obtain transcript reads. The resulting reads were processed using our in-house analysis pipeline to obtain transcript abundance. Genes that showed significant change of expression (normalized to DMSO-treated control samples) were identified (with false discovery rate < 0.05, 1.5-fold) and subjected to pathway analysis, using tools including PANTHER (v14.0, http://pantherdb.org/) and Enrichr (http://amp.pharm.mssm.edu/Enrichr/). The iLINCS database (http://www.ilincs.org/ilincs/signatures/main/upload) was used for comparison analysis to identify perturbations that produced expression profiles similar to those produced by our small molecules.

### Lifespan assays in C. elegans

Given the caveat of lifespan-assay variations for *C. elegans* studies, lifespan analysis was carried out using several different culture conditions (in liquid and on plates, in different food concentrations, using live or UV- & kanamycin-treated bacteria). Molecules were analyzed at the highest dose (∼60 μM, 0.3% DMSO, as a higher DMSO concentration has been reported to extend lifespan of *C. elegans*). Liquid culture-based lifespan assays were performed, following the protocol of others (Solis and Petrascheck 2011). Briefly, synchronized L1s of wild-type animals were fed ampicillin-resistant OP50 bacteria and treated with small molecules (∼60 μM final concentration, 0.3% DMSO) at the young-adult stage. FUDR was used at 100 μM final concentration at the L4 stage to block progeny production. The molecules were analyzed in 96-well plates, with 4 wells for each small molecule. Multiple control wells with DMSO (0.3% final concentration) were included on each plate. Small molecules were also analyzed for their ability to extend lifespan on solid agar, as described by others (Cabreiro *et al.* 2013). Hypochlorite-synchronized temperature-sensitive sterile mutants, CF4059, [*fer-15(*rrf-3*) (**b26**)II **rol-6**(**su1006**)II*; *fem-1**(**hc17**)IV*], were raised on 10-cm agar plates seeded with OP50 bacteria at 25°. Sterile day-1 adults were transferred onto mini-plates, which were seeded with normal OP50 bacteria (UV-irradiated, kanamycin-treated) and supplemented with small molecules (∼60 μM final concentration, 0.2% DMSO). Worms were scored every other day. Cumulative survival was analyzed using the STATA software (log-rank test).

Additional methods are described in Supplemental Text: the screen flow with technical details, the CdCl_2_-resistance assay, the DPPH assay of ROS scavengers, the AmplexRed assay of hydrogen peroxide, the assay of PARP inhibition, the analysis of small molecules’ effects on cell confluency, DNA-damage markers, mTOR activity and poly(Q) toxicity, plus microarray analysis and qPCR analysis. The H_2_O_2_-stress assay and analysis of pathway-activity reporters in *C. elegans* and methods for *C. elegans* RNAi are also included in Supplemental Text.

### Data availability

Table S1 shows the chemical identities of 209 primary hits, plus 61 top hits and 32 core-set hits analyzed. Table S2 shows the cell death-imaging analysis to confirm protective effects of small molecules against H_2_O_2_. Table S3 shows that certain small molecules also protect cells from heavy metal cadmium. Table S4 indicates the effects of small molecules on DNA damage-associated markers and ATP contents upon prolonged incubation. Table S5 lists the significant genes identified by RNA-seq (for Gr-4D) and microarrays (for other molecules). Table S6 shows the pathway analysis results. Table S7 shows that certain molecules might inhibit mTOR. Table S8 summarizes the effects of small molecules on *C. elegans*’ lifespan. Table S9 lists potential targets for our small molecules.

Figure S1 shows increased oxidative-stress resistance upon *AKT1* or *KEAP1* knockdown. Figure S2 shows Z-prime scores across the screen. Figure S3 indicates the analysis to exclude ROS scavengers. Figure S4 shows no obvious quenching of H_2_O_2_ by small molecules *in vitro*. Figure S5 indicates the long-term effects of small molecules on ATP levels and cell confluency. Figure S6 shows increased cell viability upon H_2_O_2_ treatment by small molecules in the RNA-seq and microarray experiment, as well as *NRF2* non-dependency for Gr-4D. Figure S7 displays the effects of small molecules on gene expression of WI-38 cells. Figure S8 indicates the analysis to identify PARP inhibitors. Figure S9 shows protective effects of certain small molecules against poly-Q toxicity. Figure S10 shows the effects of Gr-4D on *C. elegans* expressing reporters of genes related to pathways known to influence longevity, as well as the RNAi experiment to address the *sesn-1* dependency of the lifespan extension produced by Gr-4D.

The RNA-seq data for human WI-38 cells treated with the chalcone Gr-4D can be found at the GEO with the accession number GSE141675. Supplemental material available at figshare: https://doi.org/10.25387/g3.11292143.

## Results

### Small-molecule screen for oxidative-stress resistance

Primary skin fibroblasts from long-lived mouse mutants and long-lived animal species exhibit cellular resistance to the stressors hydrogen peroxide, cadmium and methyl methanesulfonate (MMS) (Harper *et al.* 2007; Harper *et al.* 2011; Salmon *et al.* 2005). For our screen, we used the WI-38 human primary-fibroblast cell line. Tumor cells were avoided because conditions that extend animal lifespan generally inhibit tumor cell growth (Kalaany and Sabatini 2009; Pinkston *et al.* 2006; Cheng *et al.* 2014). We note that not all types of cells derived from long-lived and stress-resistant animals are stress-resistant in culture. Some, such as hepatocytes, are more likely to undergo apoptosis (Kennedy *et al.* 2003). Likewise, human mammary epithelial cells incubated in IGF-1-deficient serum from humans defective in growth-hormone receptor are sensitized to die when exposed to hydrogen peroxide (Guevara-Aguirre *et al.* 2011).

**Figure 1:**
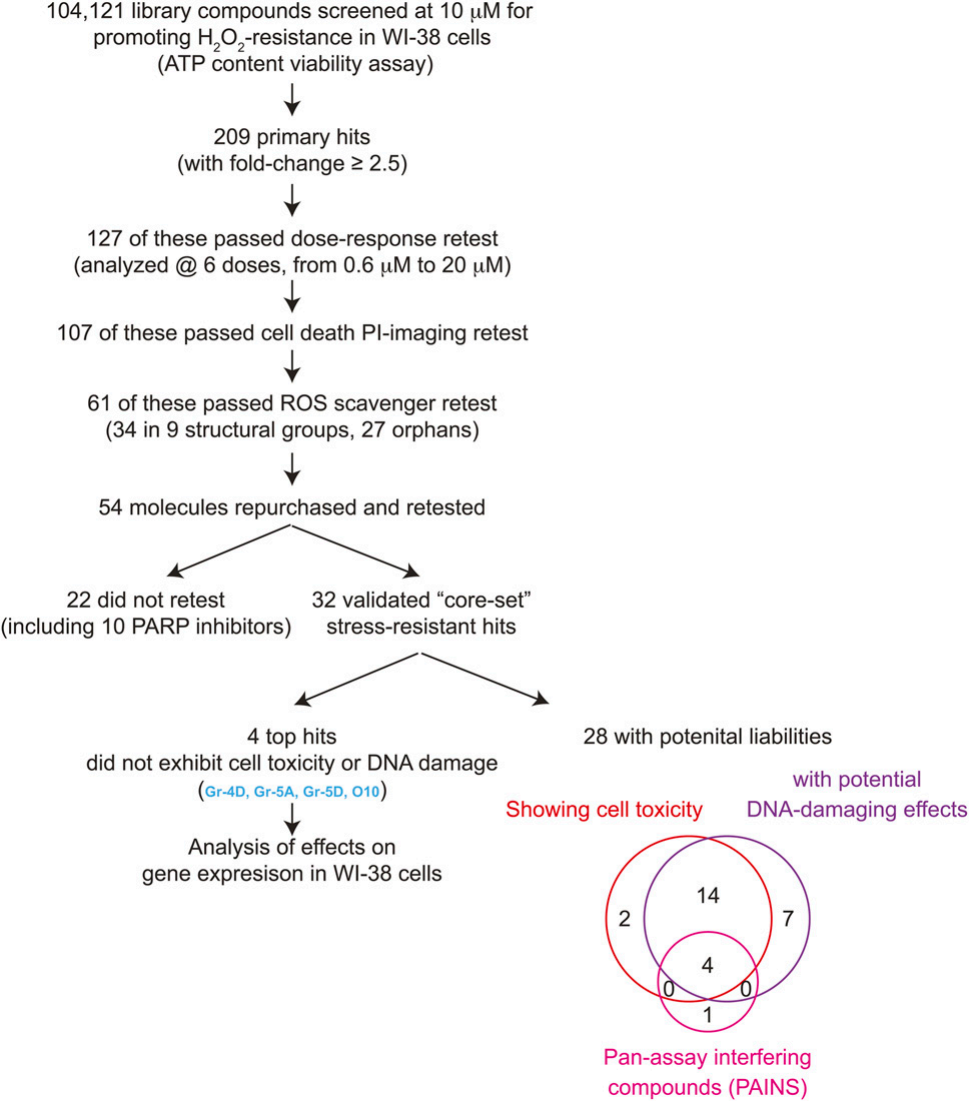
Summary of the small-molecule screen hits. A cell viability assay was performed for each of 104,121 compounds analyzed, by measuring ATP content, for WI-38 cells that were pre-incubated with 10 μM small molecules overnight and then treated with H_2_O_2_ for 3 hr. 209 primary hits were identified and subjected to a series of retests to eliminate false positives and ROS scavengers to yield the top 61 selected hits. Of these, a core set of 32 small molecules were validated and further analyzed in cells and in worms. Many molecules showed cell toxicity and potential DNA-damaging effects, and 4 top non-toxic molecules showing no liabilities were analyzed for their effects on gene expression in WI-38 cells.

In the screen, we looked for small molecules that increased cellular resistance to hydrogen peroxide (Figure 1). The screening flow is summarized in Figure 1 and detailed in Materials & Methods. Briefly, we screened a compound collection that contains 104,121 structurally diverse small molecules selected to maximize the coverage of chemical space. In our primary screen, we assessed cell viability by measuring ATP content. We selected a 3-hour treatment with 700 μM H_2_O_2_, as, importantly, small interfering RNAs (siRNAs) against the insulin/IGF-1 signal-transducing gene *AKT1* and the NRF2 inhibitor gene *KEAP1* both increased stress resistance under these conditions (Supplemental Figure 1), suggesting that we could recover the types of perturbations known to increase lifespan in animals. Altogether, we identified 209 small molecules (0.2% of molecules tested, at 10 μM) that produced signals at least 2.5-fold greater than the DMSO-treated negative control (Supplemental Table 1). We retested these candidates at six different concentrations (0.6 μM to 20 μM) and confirmed 127 that consistently produced protective effects against H_2_O_2_ at one or more of these doses (data not shown).

To exclude molecules that did not increase stress resistance but instead somehow elevated cellular ATP levels, we also carried out a secondary imaging assay for cell viability. Using propidium iodide (PI), a cell non-permeable dye that stains DNA in late-apoptotic/necrotic cells when membrane integrity is lost, we identified 107 compounds that reduced the percentage of dead cells following H_2_O_2_ stress. Predictably, among the molecules that increased ATP levels but produced little or no protective effect in PI-imaging assays were several inhibitors of poly ADP-ribose polymerase (PARP) (see Supplemental Text). PARP consumes ATP to repair DNA damage; thus, its inhibition increases ATP levels without protecting cells from oxidative stress.

**Figure 2:**
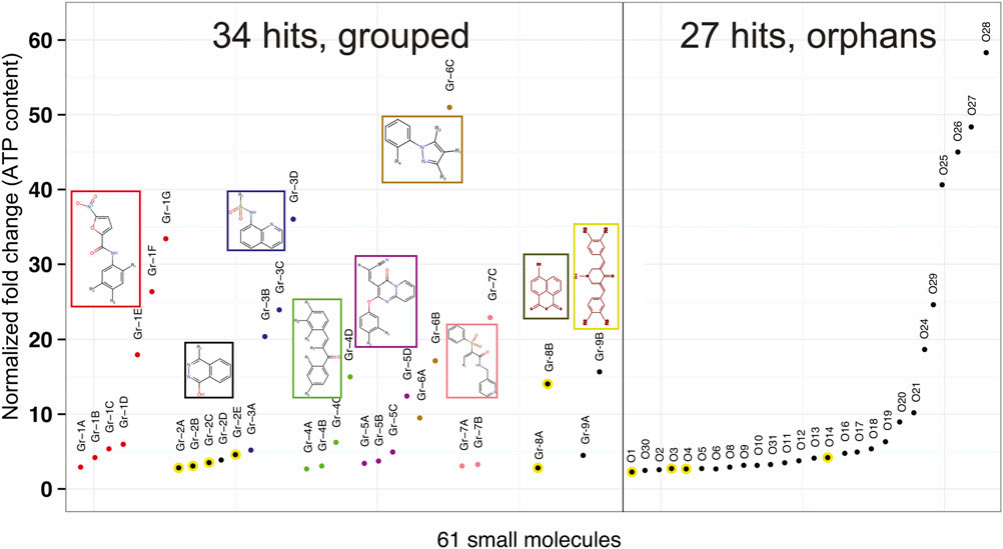
Summary of the top 61 hits that promote H_2_O_2_-resistance. Shown are normalized fold changes (ATP content) of H_2_O_2_-treated WI-38 cells, which were pre-incubated with each of the top 61 hit compounds that passed the initial selection criteria (see Figure 1). 34 molecules fell into one of nine groups (Gr-1 to 9, shown in different colors – core structures are shown) (see Supplemental Table 1 for structures). Each group comprised two or more members that share a similar core structure. In addition, we identified 27 orphan compounds. Yellow circles: PARP inhibitors (see Supplemental Figure 8).

Among these 107 hits were 32 potential derivatives of 8-hydroxyquinoline (8-HQ), a well-known reactive oxygen species (ROS) scavenger that can protect cells from H_2_O_2_ stress (Wang *et al.* 2010). We excluded additional such compounds using an *in vitro* ROS scavenger assay (Supplemental Figure 3), leaving 61 primary hits (Figure 2; Supplemental Table 1). Of these, 27 had unique chemical structures (orphans), whereas 34 fell into one of nine structural classes containing either two or more members (Gr1 to Gr9), suggesting that they may act on common targets to protect cells from H_2_O_2_. These structures are shown in Supplemental Table 1.

We were able to obtain a fresh batch of 54 of the 61 compounds and verified their molecular masses, with 3 exceptions. However, we noted that, among these 51, several batches of compounds that we continued to analyze (Gr-5A, Gr-5B, Gr-6B, Gr-6C and O27) contained a second LC-MS peak, likely a modified species. We first verified, by performing an *in vitro* Amplex Red assay, that these molecules did not quench H_2_O_2_ (Supplemental Figure 4), excluding this as a possible explanation for their protective effects. We then confirmed in multiple independent experiments, by measuring ATP content and performing additional PI-imaging, that 32 molecules (excluding all the PARP inhibitors, some of which scored borderline in the PI-imaging assays) reproducibly protected WI-38 cells during H_2_O_2_ treatment (Supplemental Table 1 & 2). Hence these 32 small molecules became our “core set” of hits for further characterization (Table 1).

**Table 1.** Summary of core-set small molecule hits by their phenotypic assay scores. 32 core-set hits, including 24 that belong to 7 structural groups and 8 orphans, were analyzed in multiple longevity-correlated assays and their behaviors are shown in this table. Their relative effects in each assay are indicated (also see Supplemental Table 1). Shown first are four top hit compounds showing no obvious cell toxicity. Asterisk (*): five molecules with signature structures of pan-assay interference (PAIN) compounds. Small molecules’ relative effects in each assay are indicated by signs. Nine molecules extended *C. elegans’* lifespan in at least 3 to 4 trials (++). N.D. not determined. Besides, 22 of the 54 repurchased compounds are not shown because: 1) Two orphan compounds (O19 and O25) scored negatively in all tests, even the hydrogen peroxide retest, and were discarded. 2) For three molecules (Gr-6A, O12 and O17), their masses did not match the predicted values by LC-MS analysis. 3) The other seventeen, including all group-2 compounds that were confirmed to be PARP inhibitors, increased ATP content upon stress (and potentially are still interesting), yet did not score positively for the cell death-imaging assay.

	Cell-based assays						*C. elegans*-based assays		Liabilities	
ID	H_2_O_2_-resistant	CdCl_2_-resistant	Potential FOXO3 Activation (by qPCR assay)	Potential NRF2 Activation (by qPCR assay)	mTOR down-regulation	Protection from Huntington's poly-Q toxicity	H_2_O_2_-resistance in *C. elegans*	Lifespan-extending scores in *C. elegans*	Potential DNA-damaging	Potential toxicity based on ATP and confluency assays
Top, non-toxic molecules (4)										
Gr-4D	yes	++	no	++	no	no	+	+++	no	no
Gr-5A	yes	++	no	+	no	no	+	no	no	no
Gr-5D	yes	no	no	+	no	no	no	no	no	no
O10	yes	++	no	no	no	no	no	no	no	no
Others with potential liability (* potential PAINS, shown at the bottom) (28)										
Gr-1A	yes	+/?	no	no	no	+	+	no	+	no
Gr-1B	yes	++	no	no	no	no	+	+	+ or no	no
Gr-1C	yes	++	no	no	no	+	no	+	+	no
Gr-1D	yes	++	no	no	no	no	no	+	+	no
Gr-1E	yes	++	no	no	no	no	no	++	+	+
Gr-1F	yes	++	no	no	no	no	no	+	+	+
Gr-1G	yes	++	no	no	no	no	no	+	+	+
Gr-3A	yes	++	no	no	no	no	no	++	+	no
Gr-3B	yes	++	no	no	no	no	+	++	+	+
Gr-3C	yes	no	no	no	no	no	no	++	+	+
Gr-4A	yes	++	no	++	no	no	no	no	+ or no	+
Gr-4B	yes	++	no	++	no	no	no	+	no	+
Gr-4C	yes	no	no	++	no	no	no	no	no	+
Gr-5B	yes	no	no	+	no	no	no	no	+ or no	no
Gr-6B	yes	++	no	+	no	+	no	no	+ or no	+
Gr-7A	yes	N.D.	+	no	+	no	++	++	+	+
Gr-7B	yes	N.D.	no	no	+	no	+	no	+	+
Gr-7C	yes	no	+	+	+	+	no	no	+ or no	+
O11	yes	++	no	no	no	no	+	+	+ or no	+
O13	yes	+/?	no	+	no	no	+	+++	+ or no	+
O18	yes	no	no	+	no	no	no	+	+ or no	+
O20	yes	++	no	+	no	no	no	+	+ or no	no
O27	yes	++	no	no	+	no	no	−	+	+
O6 (* non-toxic)	yes	++	no	+	no	no	no	+	no	N.D.
O21 (*)	yes	no	no	no	no	no	no	no	+	+
Gr-6C (*)	yes	no	no	no	no	no	no	++	+	+
Gr-9A (*)	yes	N.D.	no	+	no	no	++	+	+ or no	+
Gr-9B (*)	yes	N.D.	+	no	no	no	+	++	+ or no	+

A second cellular stress that correlates well with organismal longevity is resistance to cadmium, which also produces ROS (Harper *et al.* 2007). Therefore, we analyzed the ability of our core-set small molecules to protect WI-38 cells from cadmium, using the same ATP assay. At least 18 molecules increased resistance to cadmium as well as H_2_O_2_ (in 2 of 2 trials, Supplemental Table 3). Furthermore, 22 validated hits also protected human primary dermal fibroblasts (isolated from the skin of multiple donors) from H_2_O_2_ (in 2 of 2 trials, Supplemental Table 2), demonstrating that their protective capacity was not limited to the WI-38 cell line. We also asked whether any molecules could increase resistance of cells to the DNA-damaging agent MMS, but none of our 32 core-set hits scored positively in this assay.

Finally, we sought to eliminate small molecules that had obvious toxicities at the lowest efficacious dose. To this end, we analyzed long-term effects of the core-set molecules on ATP levels and cell confluency in culture (see Supplemental Text; Supplemental Figure 5 & Supplemental Table 4), and we also tested their ability to induce proliferation arrest or DNA damage-associated markers in the absence of H_2_O_2_ (Supplemental Table 4). We found that 25 of our 32 core-set small molecules elicited signs of DNA damage; 20 of these also produced cell toxicity (assayed either by effects on ATP content or cell confluency) (Table 1). However, five molecules exhibited none of these potential liabilities: Gr-4D, Gr-5A, Gr-5D, O6 and O10 (see Supplemental Figure 5).

Electrophilic “pan assay interference compounds” (PAINS), due to their reactive nature, can produce non-specific effects in a chemical screen (Baell and Holloway 2010; Baell and Walters 2014) (See Supplemental Text for more discussions). Of our 32 core hits, one non-toxic compound, O6, fit the criteria of a PAIN, as did 4 of the toxic compounds. Because of their relatively attractive features for drug development, the 4 non-toxic, non-PAIN molecules (Gr-4D, Gr-5A, Gr-5D and O10) were investigated most extensively in this study. However, because the toxicity we observed with the other compounds could potentially be due to off-target effects, and, interestingly, because low levels of some toxic agents (such as paraquat) have been shown to induce protective responses that extend life, we ran several additional tests on all 32 compounds.

### Effects of hit compounds on gene expression

To gain insight into potential mechanisms by which the small molecules promoted oxidative stress-resistance, we performed RNA-seq analysis on our most promising hit, the chalcone Gr-4D, which extended the lifespan of *C. elegans* up to 50% (described below; see Figure 4 & Supplemental Table 8) and carried out microarray analysis of additional hits.

**Figure 3:**
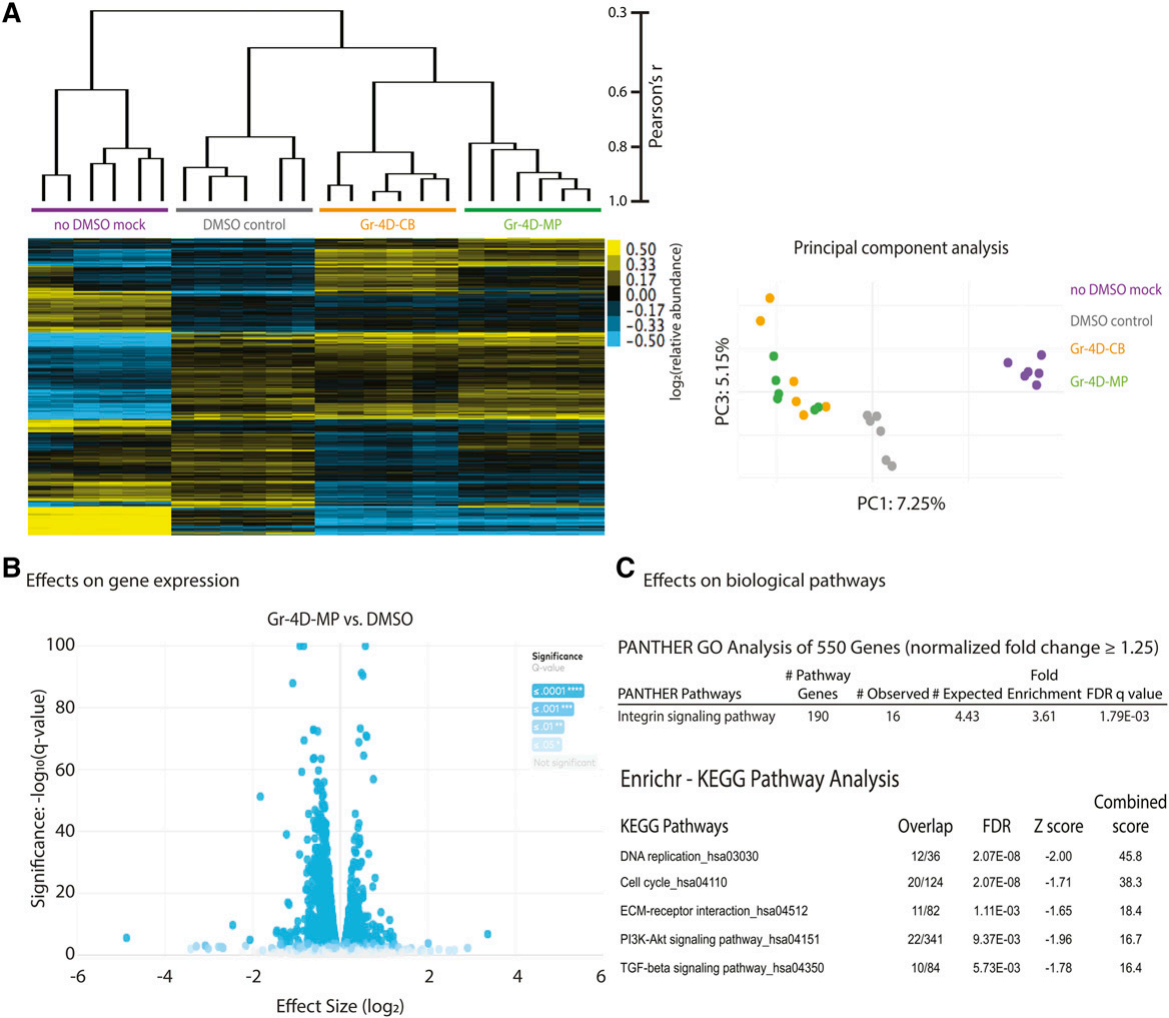
Effects of Gr-4D on gene expression of WI-38 cells. A) Pearson’s correlation between global transcriptional profiles for cells treated with Gr-4D. Shown are the normalized gene expression matrices (transcripts per million, TPM values) of the 8,000 genes detected in every RNA-seq sample, grouped by unsupervised clustering using Pearson correlation coefficient as a distance metric. Note that most DMSO (0.1%) controls were clustered together on the tree, as were the no-DMSO mock controls or samples treated with Gr-4D (2.5 μM) from two different vendors (CB, ChemBridge; MP, MolPort). This correlation was observed also in the principal component analysis (PCA), by projecting two major components (PC1 and PC3), which explained 7.25% and 5.15% variation of global gene expression, respectively. B) Volcano plot showing effects of Gr-4D on gene expression. Shown are the normalized expression level (X axis) and FDR-adjusted significance (Y axis) for one Gr-4D (from MolPort). Each dot represents a gene that was differentially expressed in WI-38 cells upon treatment with Gr-4D (see Supplemental Table 5). C) Pathway analysis indicating effects of Gr-4D on certain pathways. Shown are the top pathways affected (by PANTHER analysis of 550 significant genes, with FDR-adjust q value of overlap with the known pathway genes < 0.05; or by Enrichr-KEGG pathway analysis, with a ranking score combining both FDR and z score > 10) (see Supplemental Table 6A).

Unsupervised hierarchical clustering of the Gr-4D RNA-seq data (which considers global expression variations) showed that, as expected, biological repeats were closely correlated for each condition (Figure 3A). Similarly, this correlation was observed for controls or Gr-4D-treated samples in principal component analysis (Figure 3A). Gr-4D caused rather small expression changes for most genes. We identified 115 genes whose expression was altered by at least 1.5-fold (*vs.* DMSO control, false discovery rate < 0.05) consistently in cells treated with Gr-4D molecules from two different vendors (Figure 3B & Supplemental Table 5A). Analysis of these genes did not suggest an obvious enrichment of genes involved in specific biological pathways. When we expanded our analysis to 550 significant genes whose expression showed a modest change (≥ 1.25-fold), we observed an enrichment of genes involved in integrin signaling, including multiple collagen-encoding genes whose expression was down-regulated, suggesting an attenuation of this pathway (Figure 3C & Supplemental Table 6A). Further analysis using the Enrichr suite, which integrates multiple types of pathway analysis tools, suggested a significant enrichment of genes involved in DNA replication, cell cycle regulation, PI3K/AKT and TGF-beta signaling (Figure 3C & Supplemental Table 6).

Among the 550 genes modestly changed upon Gr-4D treatment, we did not observe a strong signature for the longevity-associated gene *FOXO3*, but we did observe a modest induction of a number of NRF2 target genes, including nuclear receptor *NR0B1*, NAD(P)H dehydrogenase *NQO1*, aldo-keto reductases *AKR1B10* and *AKR1C1* (Supplemental Table 5A). This was noteworthy, because NRF2/SKN-1 is a key regulator of oxidative stress and xenobiotic phase 2 detoxification, and it can extend animal lifespan (Tullet *et al.* 2008; Sykiotis and Bohmann 2008). We also asked whether Gr-4D’s expression profile resembled any signatures identified under other conditions in the iLINCs database, including gene knockdowns and small-molecule perturbations. Interestingly, *KEAP1* knockdowns, which activate NRF2, scored at the top among the genetic perturbations that produced profiles significantly resembling Gr-4D’s signature (Supplemental Table 6). These results suggested that Gr-4D could elicit a modest activation of NRF2, consistent with its identity as a chalcone (see Properties of Hit Molecules), compound known to activate NRF2.

We also noted that inhibitors of heat shock protein 90 (HSP90) and histone deacetylases (HDACs), as well as inhibitors of the proteasome, were among the top chemical perturbations that produced gene expression signatures showing very strong and positive correlations to Gr-4D’s (Supplemental Table 6A). HSP90 is known to form a complex with aryl hydrocarbon receptor (AhR), a ligand-activated transcription factor that regulates genes such as xenobiotic metabolizing enzymes (Prodromou 2016). Dissociation of HSP90 upon xenobiotic stimulation could facilitate activation of AhR and enhance NRF2 activity in response to oxidative and xenobiotic stress (Tan and Wahli 2014). Interestingly, HDAC inhibitors, which could cause hyperacetylation and inhibition of HSP90 (Kovacs *et al.* 2005), protected neurons against oxidative stress-induced cell death *in vitro* and *in vivo* (Langley *et al.* 2005). Finally, the proteasome inhibitor MG-132 induced nuclear translocation of NRF2 in human vascular endothelial cells (Sahni *et al.* 2008), and Nrf2-dependent induction of proteasome and immunoproteasome promoted adaptation to oxidative stress in mouse embryonic fibroblasts (Pickering *et al.* 2012). Consistent with these findings, we observed that siRNA knockdown of several proteasome subunits enhanced resistance of WI-38 cells to H_2_O_2_.

We also analyzed gene expression by microarray analysis of WI-38 cells treated with 3 other non-toxic molecules (Gr-5A, Gr-5D and O10). For comparison, we included other members of the structural group 4, which produced long-term toxicity. We also included the other group-5 molecule Gr-5B, which, despite its modest induction of TP53BP1, did not appear to exert obvious cell toxicity.

The toxic molecules produced numerous gene-expression changes, but, like Gr-4D, the non-toxic hit compounds induced relatively few significant gene changes (typically less than 500) in the absence of H_2_O_2_ [using Statistical Analysis of Microarrays (SAM) (Tusher *et al.* 2001), which focuses on highly affected changes] (Supplemental Table 5B). Unsupervised hierarchical clustering of the global gene expression profile showed that biological repeats were also closely correlated, as were the multiple rapamycin-treatment controls (Supplemental Figure 7). Similarly, profiles for the toxic group-4 members also resembled one another and formed a distinctive cluster. Unlike with SAM, using this method, we found that group-5 molecules (Gr-5B & Gr-5D) that share a core structure were loosely correlated with each other. None of the other compounds clustered together.

We performed SAM to identify genes that showed significant changes for each condition (Supplemental Table 5B), and further analyzed their effects on biological systems using pathway analysis tools. Our pathway analysis indicated that, for example, the non-toxic Gr-5A molecule affected genes involved in prostaglandin biosynthesis (Supplemental Table 6). The other group-5 molecule Gr-5D, despite its structural similarity to Gr-5A, affected genes involved in response to DNA damage and stress, as suggested by the downregulation of multiple genes encoding histones and the up-regulation of genes encoding heat shock proteins. The orphan molecule O10 appeared to induce the integrated stress response (ISR) mediated by ATF4 and PERK, as suggested by the up-regulation of the ISR regulator *CHOP*/*DDIT3*, asparagine synthetase *ASNS* and insulin like growth factor binding protein 1 *IGFBP1*, which is known to be induced by ATF4 upon ER stress (Marchand *et al.* 2006). Interestingly, ATF4 is up-regulated in long-lived mutant mice (Li *et al.* 2014). Finally, gene changes induced by the toxic group-4 molecules were strongly and consistently enriched within the NRF2-mediated oxidative stress-response pathway, as suggested by the induction of multiple antioxidant and xenobiotic stress response genes (Supplemental Table 5B & 6B).

### Effects of hit compounds on known longevity pathways

In addition to analyzing gene expression in cells treated with our 4 best stress resistance-inducing molecules, we also examined all 32 hit compounds from our screen in cellular assays relevant to the biology of aging. Specifically, we asked whether they could activate proteins known to extend lifespan, inhibit mTOR signaling, and/or attenuate toxic protein aggregation (see Supplemental Text for protein aggregation data).

#### NRF2:

In our microarray experiments, a clear signature of NRF2 activity, detected using pathway analysis, was observed only for toxic compounds. To address this issue more broadly, we treated cells with all 32 core-set small molecules and used RT-qPCR to analyze the expression of several canonical NRF2-regulated genes, including *HMOX1* [heme oxygenase (decycling) 1; an anti-oxidant], *NQO1* [NAD(P)H dehydrogenase, quinone 1; a phase 2 detoxification enzyme], *GCLC* (glutamate–cysteine ligase, catalytic; a glutathione-synthesis enzyme) and *GSTM1* (glutathione S transferase) (Hur and Gray 2011; Suzuki *et al.* 2013).

Of the 4 best hits described above (Gr-4D, Gr-5A, Gr-5D and O10), three (Gr-4D, Gr-5A and Gr-5D) induced the expression of two or more NRF2-regulated genes at least modestly (by more than 1.5-fold; Supplemental Table 1), suggesting potential NRF2 activation. In addition to these 3 non-toxic compounds, 11 of the remaining 28 toxic or non-specific compounds also appeared to activate NRF2. Of these, Gr-9A induced *HMOX1* by almost 50-fold in WI-38 cells, and this compound also protected *C. elegans* from hydrogen peroxide (in 2 of 2 trials) (Supplemental Table 8). Consistent with the microarray analysis (Supplemental Table 5B), we also noted that all the toxic group-4 molecules resulted in significant induction of NRF2 target genes. These data suggested enrichment for NRF2-activating small molecules in our screen, which is expected, as we selected for agents that could promote oxidative stress-resistance.

NRF2 activation could be one possible mechanism by which our small molecules promote oxidative-stress resistance. In this case, we would expect that *NRF2* to be required for the increased resistance. To address this question, we knocked down *NRF2* by more than 95% in WI-38 cells via siRNA transfection (Supplemental Figure 6A). Compared with *NRF2(+)* cells, *NRF2*-deficent cells showed a high level of propidium iodide staining, and the fraction of PI-positive cells increased further upon treatment with Gr-4D in a dose-dependent manner even in the absence of H_2_O_2_, likely reflecting increased xenobiotic sensitivity upon the loss of *NRF2* (Supplemental Figure 6B). To compensate for this sensitivity to oxidative stress, we treated the *NRF2*-deficient cells with a lower dose of H_2_O_2_ (500 μM instead of 700 μM), finding that Gr-4D could protect *NRF2(+)* cells under this condition. As expected, compared with *NRF2(+)* cells, *NRF2*-deficient cells were more prone to die upon treatment with H_2_O_2_. To our surprise, Gr-4D (at 3 different doses) was still quite protective, significantly reducing the fraction of PI-positive cells (Supplemental Figure 6B). Thus, *NRF2* was not required for this chalcone to promote oxidative-stress resistance. Gr-4D must act, at least in part, through a different, *NRF2*-independent mechanism.

#### FOXO3:

Many perturbations that increase lifespan and stress resistance in animals do so in a *FOXO*-dependent fashion (Kenyon 2010). None of our “four-best” compounds scored positively for potential FOXO3A activation. However, 3 toxic compounds, including 2 group-7 molecules, could up-regulate the expression (by more than 1.5-fold) of at least 2 of the 5 FOXO3A-regulated genes analyzed: *SOD2* (superoxide dismutase), *GADD45A* (cell cycle regulator), *CAT* (catalase), *DDB1* (damage-specific DNA binding protein) and *TXNIP* (thioredoxin interacting protein) (Supplemental Table 1).

#### Sestrin:

Sestrin genes are regulated by FOXO3 (Nogueira *et al.* 2008), NRF2 (Shin *et al.* 2012) and p53 (Budanov *et al.* 2004). Through direct effects on anti-oxidant peroxiredoxins and through the AMPK and mTOR pathways, sestrins can suppress ROS production and protect cells from oxidative stress, transformation, and genomic instability (Budanov and Karin 2008). Many studies have indicated that sestrins could be pro-longevity factors. RNAi inhibition of the sestrin gene *sesn-1* has been shown to shorten lifespan, while its overexpression promotes longevity of *C. elegans* (Yang *et al.* 2013). Loss of *Drosophila dSesn* has been shown to lead to age-associated pathologies, including fat accumulation, mitochondrial dysfunction, muscle degeneration, and cardiac malfunction, which could be blocked by pharmacological activation of AMPK or inhibition of TOR (Lee *et al.* 2010). Likewise, sestrin deficiencies in mice exacerbated obesity-induced diabetic conditions (Lee *et al.* 2012; Lee *et al.* 2013). Furthermore, sestrins have been shown to activate Nrf2 and, by promoting p62-dependent autophagic degradation of Keap1, prevent oxidative damage in the liver of mice (Bae *et al.* 2013). More recently, the Sabatini group showed that sestrin 2 is a leucine sensor, and that leucine can disrupt sestrin-GATOR2 interaction and result in mTORC1 activation (Wolfson *et al.* 2016). In our study, of the 32 core-set hits, at least 9 molecules were found to induce the expression (more than 1.5-fold) of *SESN1* (Supplemental Table 1).

Among the four non-toxic compounds, only Gr-4D scored positively for *SESN1* induction. In addition to Gr-4D, 8 of the 28 compounds with liabilities also induced *SESN1*. We noted that these compounds with liabilities, but not Gr-4D, also scored positively for the DNA damage-markers γH2A.X and TP53BP1, and genotoxic stress previously has been shown to up-regulate sestrins through the induction of p53 (Budanov and Karin 2008). Interestingly, both p53 and Nrf2 are highly expressed in the long-lived, stress-resistant naked mole-rat and may contribute to its longevity, and at least in the case of p53, possibly also reduce its risk of cancer (Lewis *et al.* 2012). In this regard, sestrin-inducing molecules could be useful for promoting healthy aging.

#### mTOR inhibition:

Mammalian target of rapamycin (mTOR) is a crucial regulator of cell growth and metabolism and has been implicated in aging and many diseases, including cancer, diabetes and neurological diseases (Zoncu *et al.* 2011; Dazert and Hall 2011). This connection has stimulated interest in developing novel mTORC1 inhibitors (Benjamin *et al.* 2011). In addition to rapamycin and its analogs, the anti-diabetes drug metformin (Kalender *et al.* 2010), as well as other AMPK activators such as 2-deoxy-D-glucose (Inoki *et al.* 2003) and AICAR (Shaw *et al.* 2004), can also inhibit mTORC1.

We examined the effects of our 32 core-set molecules (at three doses, 5 μM, 10 μM and 20 μM) on the phosphorylation status of ribosomal protein S6 (RPS6), a target of mTORC1, and asked whether they could potentially inhibit mTOR. Rapamycin, as a control, reduced the normalized ratio of phosphorylated RPS6 (p-RPS6) by ∼95%.

None of the non-toxic compounds appeared to inhibit mTOR. However, we found that 4 toxic molecules (all 3 members of the 7^th^ structural group and O27) reduced the normalized ratio of p-RPS6 (∼30–60%, in at least 2 of 3 trials, Supplemental Table 7); note that O27 also modestly induced sestrin, a known inhibitor of mTORC1.

### Effects of hit compounds on C. elegans

#### Hydrogen peroxide resistance:

We analyzed our 32 core hits and found that 2 small molecules (Gr-7A & Gr-9A) increased *C. elegans*’ resistance to a lethal dose of hydrogen peroxide (in 2 of 2 trials, Supplemental Table 1 & 8). Both of these molecules caused toxicity in cultured mammalian cells. We noted that compounds that scored negatively in *C. elegans* are not necessarily uninteresting. As these molecules were identified in a human cell-based screen, their bioavailability or target engagement could be different in worms.

#### Lifespan extension:

Because most conditions that extend *C. elegans*’ lifespan increase stress resistance, we analyzed how our small molecules affected lifespan. To ensure robustness, we used several different assay conditions (culturing in liquid, on OP50 bacteria-seeded agar plates, on live or UV-irradiated bacteria, and using FUDR-treated or genetically induced sterile animals).

**Figure 4:**
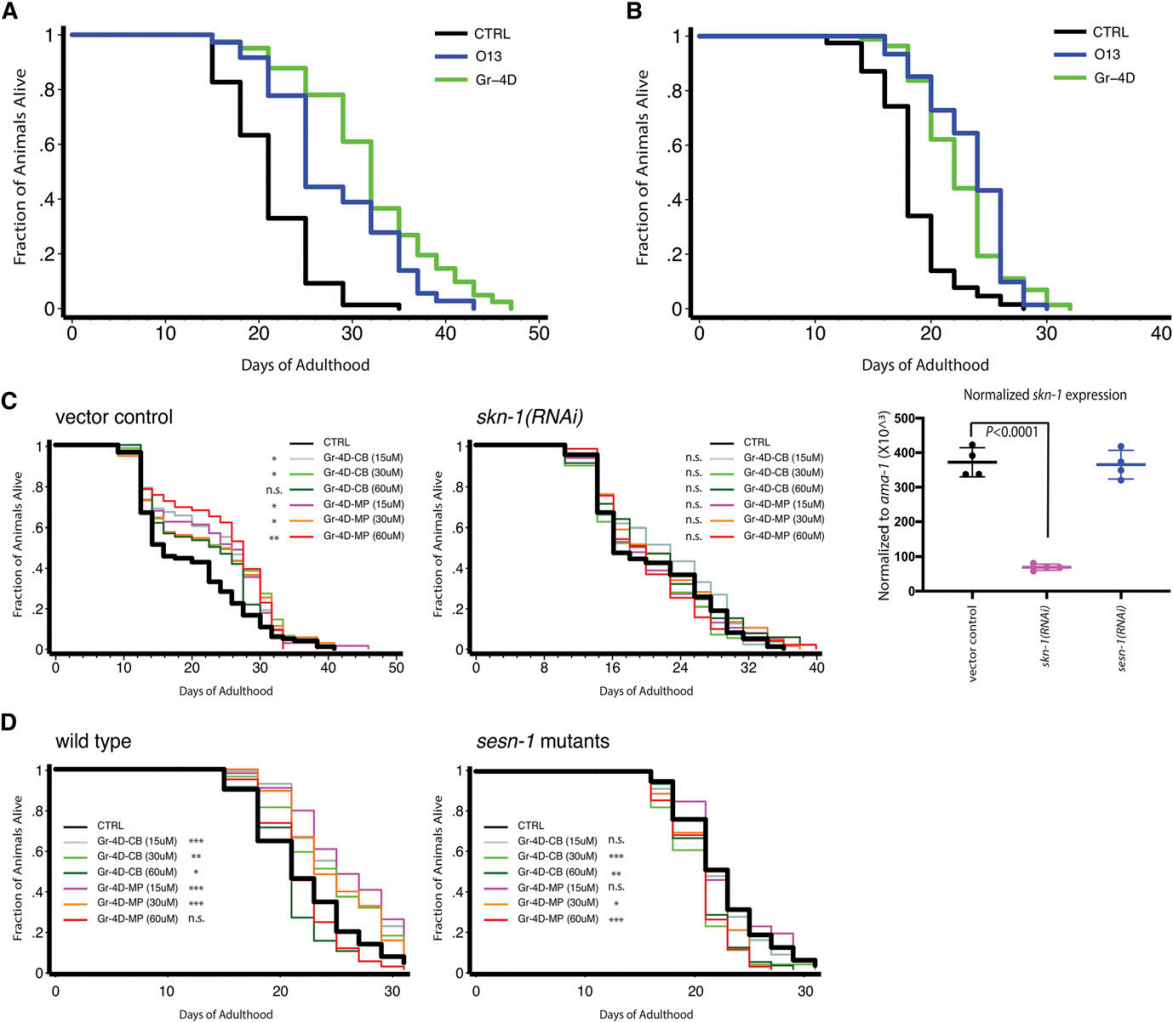
Extension of *C. elegans*’ lifespan by small molecules. Small molecules were analyzed for their ability to extend the lifespan of *C. elegans*. Two small molecules (Gr-4D, without cell toxicity; O13, with cell toxicity) that consistently extended lifespan in multiple independent assays are shown. A) Wild-type animals, grown in liquid, with FUDR to block progeny production, 20°C. Control, 21.2 ± 0.5 (mean ± SEM in days), n = 77/81 (observed/total); Gr-4D-treated, 31.9 ± 1.1 (50.5% increase), n = 41/41, *P* < 0.0001 (log-rank test); O13-treated, 28.1 ± 1.1 (32.5% increase), n = 36/36, *P* < 0.0001. B) Temperature-sensitive sterile mutant animals, grown on plates, without FUDR, 25°C then shifted to room temperature (∼22°C) as adults. Control, 18.4 ± 0.4, n = 67/80; Gr-4D-treated, 22.5 ± 0.4 (22.2% increase), n = 74/95, *P* < 0.0001 (log-rank test); O13-treated, 23.4 ± 0.4 (27.2% increase), n = 72/76, *P* < 0.0001 (see Supplemental Table 8 for details). C) The lifespan extension produced by Gr-4D requires *skn-1*. RNAi-sensitive mutants were fed with control vector or *skn-1* RNAi bacteria and treated with Gr-4D (from two different vendors, CB = ChemBridge, MP = MolPort) at multiple doses. In parallel, RNAi-treated animals were collected for RT-qPCR analysis, which indicated that *skn-1* mRNA level was reduced by ∼80% in these animals (normalized to *ama-1*, n = 4 each; one-way ANOVA, followed by Dunnett’s multiple comparison, *P* < 0.0001). In this experiment, Gr-4D extended lifespan of control animals by ∼15–20%. D) The lifespan extension produced by Gr-4D requires *sesn-1*. In this experiment, lower doses but not a high dose of Gr-4D extended lifespan of wild type by ∼10–17%. (The reason for this discrepancy was unclear). Log-rank test: *, *P* < 0.05; **, *P* < 0.01; ***, *P* < 0.001; n.s., not significant (see Supplemental Table 8 for details of lifespan data).

We analyzed our 32 core-set molecules, and in multiple independent experiments, found that 9 small molecules extended the animals’ lifespan in at least 3 to 4 trials (from ∼10% to ∼50%) (Supplemental Table 8). Gr-4D was the only non-toxic compound that consistently produced significant life-extending effects in 4 or more trials (average increase ∼24%; 50% in one trial) (Figure 4). Among the other 8 toxic molecules, O13, an orphan compound, also extended lifespan in a consistent manner (average increase ∼18%). Conversely, many molecules did not extend lifespan in any trial, and some even shortened lifespan, including two group-4 compounds (Gr-4A and Gr-4C) and O27 (Supplemental Table 8). In principle, lifespan might be increased due to caloric restriction; however, we did not observe an obvious reduction in pumping rates for worms treated with the lifespan-extending molecules (data not shown), and the animals were not pale, a hallmark of calorically restricted animals.

To investigate the potential mechanism by which the chalcone Gr-4D extended lifespan, we asked whether it might affect gene activities known to influence longevity. To do this, we examined its effects on the expression of pathway-specific reporters, including the ER-stress reporter *Phsp-4*::*gfp*, the mitoUPR reporter *Phsp-6*::*gfp*, the NRF2/SKN-1 target gene reporter *Pgst-4*::*gfp* (for oxidative stress), and the autophagy reporter *LGG-1*::*GFP*. We observed that Gr-4D, at a dose that extended lifespan, caused modest induction of the *gst-4* transcriptional GFP reporter (Supplemental Figure 10C), without exerting obvious effects on the others. As this glutathione transferase *gst-4* gene is up-regulated in response to SKN-1/NRF2 activation upon oxidative stress, these findings suggested that activation of SKN-1/NRF2 by Gr-4D might contribute, at least in part, to the lifespan extension in *C. elegans*.

To test directly whether *skn-1* is required for the lifespan extension produced by Gr-4D, we examined this chalcone at multiple doses in RNAi-sensitive mutants fed with control or RNAi bacteria expressing dsRNA of *skn-1*. When initiated from day 1 of adulthood, RNA interference reduced the level of *skn-1* mRNA by ∼80% in 3 days (Figure 4C). Whereas Gr-4D extended the lifespan of control animals by ∼15–20%, it failed to extend the lifespan of animals subjected to *skn-1* RNAi (Figure 4C & Supplemental Table 8). Thus, we concluded that Gr-4D required *skn-1* for promoting longevity in worms, though, in contrast, it was able to increase H_2_O_2_-resistance independently of *NRF2* in cultured human cells (Supplemental Figure 6B).

Sestrins are known to be involved in the NRF2/SKN-1-regulated stress response. Therefore, we examined whether *sesn-1*, the only worm ortholog of human sestrins, might be required for the lifespan-extending effects of Gr-4D. Upon modest reduction (∼30%) of *sesn-1* mRNA level by RNAi inhibition (Supplemental Figure 10D), Gr-4D also appeared to lose its ability to extend lifespan, even though we observed a trend of lifespan extension in animals treated with high dose of this molecule (*P* = 0.0518) (Supplemental Figure 10D & Supplemental Table 8). Given the caveat of insufficient inhibition of gene expression by RNAi, we further addressed the dependency of *sesn-1* by analyzing animals that carried a potential loss-of-function allele of *sesn-1*. We found that Gr-4D doses that extended wild-type lifespan were not able to extend the lifespan of these mutants (Figure 4D).

Taken together, these results suggested that the chalcone Gr-4D acts at least in part through the sestrin/NRF2/SKN-1 stress-response axis to extend lifespan of *C. elegans*.

## Discussion

To enrich for compounds that might slow aging, we screened for a cellular phenotype that is common to cells from many long-lived mutant animals and from naturally long-lived species of mammals and birds: resistance to oxidative stress. We screened ∼100,000 small molecules for their ability to protect a human primary fibroblast cell line from a lethal dose of hydrogen peroxide and retested the top hits for their ability to protect cells from the heavy metal cadmium and the DNA-damaging agent MMS. Many of these compounds conferred resistance to both hydrogen peroxide and cadmium. In addition, remarkably, ∼1/3 of our 32 core-set molecules extended *C. elegans*’ lifespan (see the summary in Table 1).

### Properties of hit molecules

#### Chalcones:

Four of our hits were chalcone-family members, including our most interesting hit Gr-4D, which increased lifespan consistently in worms (up to ∼50%, 4 of 4 trials) with no apparent toxicity (Figure 4, Supplemental Table 8, also see Table 1). Our 104K-compound screening library contained 71 chalcones (exact core-structure match), yet we only identified 4 among our 61 primary hits (group 4), suggesting an important role of side groups in determining H_2_O_2_-resistance (with the caveat that the others were not re-tested).

Chalcones have been reported to produce many beneficial health effects, including anti-cancer, anti-HIV, anti-malarial, anti-inflammatory and anti-allergic activities (Sahu *et al.* 2012; Batovska and Todorova 2010). They have a wide variety of molecular targets, many of which have relevance for cancer, which might explain their anti-proliferative activities against cell lines derived from many types of tumors (Yadav *et al.* 2011; Solomon and Lee 2012).

Chalcones, with a backbone of α,β-unsaturated ketone, are known to activate NRF2. In line with this, we observed consistent signatures for NRF2-regulated genes in both worms and human cells; however, genetic epistasis analysis indicated that, while NRF2 may potentially contribute to the stress resistance of human cells, NRF2-independent mechanisms play an important role as well.

A recent study has identified 4,4’-dimethoxychalcone (DMC) as a natural compound that extended lifespan in multiple species, including yeast, worms and flies (Carmona-Gutierrez *et al.* 2019). The authors proposed that lifespan extension could be attributed DMC’s ability to induce autophagy. We did not observe obvious effects of our chalcones on autophagy in mammalian cells, using the Promega LC3 HiBiT assay system (with TOR inhibitor rapamycin as a positive control), nor did we observe significant effects of DMC in promoting resistance to H_2_O_2_ in WI-38 cells. The significance of this is not clear, as, for reasons we do not understand, we were also unable to observe an increase in autophagy with DMC in our assay system.

### Reported properties of hit molecules

We referred to chemical databases (for example, PubChem) to cross-reference our small molecules to other high-throughput screens. We found that at least 9 molecules had been identified in multiple screens and suggested to affect certain human protein targets (Supplemental Table 9; See supplementary discussion). Of these, the chalcone Gr-4D, our top non-toxic compound, has been identified in many screens in the PubChem database. For example, it has been reported to kill fibrosarcoma cell lines that produce the oncometabolite 2-hydroxyglutarate (2HG) (AID 686970, with potency of ∼3 μM; see Supplemental Table 9). Other studies have suggested that Gr-4D can inhibit IL-1B-mediated inflammasome signaling (AID 743279) and activate p53 by inhibiting the MDM2/MDM4 interaction (in multiple assays). Finally, Gr-4D could induce DNA-rereplication in both SW480 colon adenocarcinoma cells and MCF 10A epithelial cells (with a potency of ∼5-6 μM), which in turn can induce the DNA damage response and apoptosis (BioAssay AID 624296 & 624297, reported target GMNN). Given the concern of genome instability that can be caused by DNA re-replication, it would be interesting to analyze whether a lower dose of Gr-4D could be tolerated *in vivo*.

Among the remaining 8 compounds with liabilities (Gr-1F, Gr-3A, Gr-3B, Gr-3C, Gr-7C, O13 and O18, all toxic; plus O6, non-toxic, yet a PAIN molecule), Gr-3B and Gr-7C were identified in multiple assays that examined different biological targets (ranging from NK-κB signaling, p53 regulation, potassium channel regulation, to protein SUMOylation). This raises a concern about non-specific actions of these molecules in cells. However, except for O6, which contains a reactive phenolic Mannich base, the other molecules do not appear to carry obvious PAINS signatures. By contrast, curcumin, a known PAIN molecule, has been found in at least 2167 assays with 92 reported protein targets (of these, 79 are human proteins, including NRF2, see Supplemental Table 9). Likewise, the other PAIN molecule resveratrol has been identified in at least 4266 assays with 91 active protein targets (these include 74 human proteins, such as SIRT1 and FOXO3).

To our surprise, despite the differences in their structures, several different molecules appeared to affect the same protein targets (Supplemental Table 9). For example, 4 molecules (the non-toxic chalcone Gr-4D and toxic Gr-1F, Gr-3B and Gr-7C) were shown previously to affect the cystic fibrosis transmembrane conductance regulator (CFTR), a chloride channel protein that is involved in multi-drug resistance (Borst *et al.* 1999). Like Gr-4D, 4 cell-toxic compounds (Gr-1F, Gr-3A, Gr-7C and O13) were also reported to target geminin GMNN, a DNA replication inhibitor. In addition, Gr-4D, Gr-3B and Gr-7C have together been identified in different studies and reported to inhibit multiple targets: for example, interleukin IL1B, a mediator of inflammasome signaling; MDM2 and MDM4, E3 ubiquitin protein ligases and negative-regulators of p53; NOD1 and NOD2, intracellular pattern-recognition receptors that regulate apoptosis and inflammation; and TDP1, a tyrosyl-DNA phosphodiesterase that interacts with PARP and regulates DNA damage response (see Supplemental Table 9). We do not know whether the ability of our molecules to promote oxidative-stress resistance is mediated, in part, by their effects on these reported target proteins.

#### Inhibition of NF-κB signaling:

Among the top non-toxic compounds, Gr-4D has been found in at least two screens linked to NF-κB signaling. In one case, Gr-4D was reported to increase the expression of a luciferase reporter driven by the NF-κB promoter in the SH-5YSY human neuroblastoma cell line (AID 1239). Curiously, it was also identified in another study as a molecule that inhibited TNF-alpha-induced NF-κB activation in HEK 293T cells (AID 1852), leaving its regulation of NF-κB in question. Among the remaining members of the 32 core-set compounds, Gr-3C was identified previously as a molecule that induced the NF-κB inhibitor NFKBIA in two screens (EC_50_, 8.5 μM and 11.0 μM; see AID 317145 and 317143), consistent with the report from another screen suggesting it as an RELA inhibitor in HUVEC cells (IC_50_, 2.0 μM, AID 317146). These two studies above also identified Gr-3A, but not Gr-3B (which has a different side group that could introduce steric hindrance), as an NFKBIA-inducing compound (EC_50_, 11.0 μM and 12.0 μM). These findings are consistent with our analysis using the *in silico* docking database ZINC (Irwin *et al.* 2012), which predicted that Gr-3C bind to both NFKBIA and RELA (data not shown).

NF-κB signaling controls cell survival, differentiation, and proliferation. Key components of the pathway mediate inflammatory responses (Lawrence 2009) and have been implicated in many diseases, including cancer, autoimmune diseases, neurodegenerative diseases, cardiovascular diseases and diabetes (Hayden and Ghosh 2012). Recently, it was shown that blocking age-related hypothalamic NF-κB activation could retard aging and extend lifespan of mice (Zhang *et al.* 2013), thus potentially highlighting a role of chronic inflammation in aging (Salminen *et al.* 2008; Jenny 2012). Of note, despite long-term cell toxicity observed for group-3 molecules, we noted that they all extended *C. elegans*’ lifespan (3 of 3 trials, ∼8–33%), even though worms do not have apparent NF-κB homologs.

### Hit molecules as regulators of longevity

In animals, the rate of aging can be influenced by many factors, including a network of signaling proteins and transcription factors that also sense nutrients, energy levels and stress. Perturbing many genes in this network can extend healthspan and increase disease-resistance and lifespan (Fontana *et al.* 2010; Bartke 2011). For example, a well-known case is the insulin/IGF-1/FOXO signaling system that regulates lifespan among many different experimental species, and likely also small dogs and bats (Kenyon 2010). *AKT1* knockdown, our control that was expected to activate FOXO3, increased stress resistance in our assay. However, we identified only 3 molecules within our 32 core-set hits that may activate FOXO3, suggesting that our stress-resistance screen may not be ideal to look for FOXO3 activators.

Conversely, we observed an enrichment of small molecules that can activate NRF2. Increased activity of SKN-1/NRF2, the oxidative stress and xenobiotic phase II detoxification regulator, can extend life independently of *daf-16**/**foxo*. We identified 14 potential NRF2 activators (3 non-toxic molecules, plus the non-toxic yet non-specific PAIN O6, as well as 10 other molecules with liabilities – see Results), of which at least 7 molecules (including 2 non-toxic, Gr-4D and Gr-5A; plus 5 other molecules with liabilities) increased resistance to both H_2_O_2_ and cadmium (note: scored as “++”). 13 of the 14 molecules activated NRF2 without affecting FOXO3 or mTOR (Table 1); the one exception, Gr-7C, was also cell-toxic. Thus, NRF2 could be a significant contributor that promotes stress resistance in our screening conditions, consistent with the finding that NRF2 is activated in fibroblasts from long-lived animals, which also are resistant to multiple stressors (Leiser and Miller 2010). That said, it was interesting that, while it might contribute to stress resistance, our epistasis analysis with *NRF2* siRNA knockdown indicated NRF2 was not required for the increased stress resistance of WI-38 cells produced by our small molecule hits.

The potential enrichment of NRF2-activating small molecules in our screen is also consistent with the anti-oxidative role of NRF2, and it may have implications for human aging as well. In human fibroblasts, reduced NRF2 function has been shown to occur in replicative senescence and *NRF2* silencing may lead to premature senescence (Kapeta *et al.* 2010). Conversely, Kapeta and co-workers also showed that molecules that activate NRF2 can enhance the survival of human fibroblasts following oxidative stress and extend their replicative lifespan. Interestingly, long-term exposure to the mTOR inhibitor rapamycin has been shown to increase mitochondria biogenesis and increase replicative lifespan of human fibroblasts, and these effects appeared to be mediated, at least in part, by p62/SQSTM1-associated degradation of KEAP1 and activation of NRF2 (Lerner *et al.* 2013). Similarly, multiple studies showed that rapamycin-induced lifespan extension requires SKN-1/NRF2 but not DAF-16/FOXO in *C. elegans* (Harrison *et al.* 2009; Wilkinson *et al.* 2012; Bjedov *et al.* 2010; Robida-Stubbs *et al.* 2012). Conversely NRF2 activation also has been linked to several human age-related diseases, including atherosclerosis, neurodegenerative diseases, and certain types of cancer (Hybertson *et al.* 2011). Small-molecule modulators of the KEAP1-NRF2-ARE pathway are under development as potential preventive and therapeutic agents (Magesh *et al.* 2012), but for NRF2 activators, whether their apparent ability to slow aging is compatible with disease resistance in humans remains to be seen.

### Hit molecules as potential cancer therapeutics

Many long-lived animal mutants are resistant to age-related diseases (Le Couteur *et al.* 2012), including cancer (Ikeno *et al.* 2009), protein-aggregation diseases (Morley *et al.* 2002; Cohen *et al.* 2009) and heart disease (Harrington *et al.* 2007; Birse *et al.* 2010; Wessells *et al.* 2004). Thus, the small molecules we seek may counter multiple age-related diseases.

Different species in nature have very different lifespans, but within each, the risk of cancer rises with age. This correlation in nature between slow aging and delayed cancer suggests that the same pathways that slow aging may antagonize cancer, and, in fact, many longevity pathways have anti-cancer activity (though some, such as Nrf2, can promote the growth of certain tumors). Importantly, these pathways inhibit a wide variety of cancers (Kalaany and Sabatini 2009), which is what we predict for our small molecules. In fact, some of our stress-resistance hits, like the chalcones, are members of structural classes with known anti-tumor activity. When we compared the transcriptional signatures of cells treated with small molecules, we noted that several group-4 and group-5 molecules appeared to attenuate basal cell carcinoma signaling, based on the expression patterns of pathway genes consistent with prior knowledge (data not shown).

Finally, even the small molecules that have cell toxicity could be interesting. In principle, any toxicity caused by these hits, which are administered at relatively high levels, could be off-target and thus dialed out via medicinal chemistry. Alternatively, they could be on-target toxicities. In the latter case, they could potentially increase stress resistance by activating cell-protective mechanisms, as do low levels of the herbicide paraquat in invertebrates, a situation (often called “hormesis”) that can extend lifespan in experimental organisms. Furthermore, toxicity could be cell type-specific. For example, we found that two group-7 molecules (Gr-7A & Gr-7B), which both produced long-term cell toxicity and appeared to inhibit mTOR, reduced the ATP content by ∼40–50% in a human lung adenosquamous carcinoma cell line HTB178 (data not shown). Conversely, when administered overnight at 10μM and assayed in parallel, these same molecules did not produce obvious effects on ATP levels in either the WI-38 primary cell line or in CRL-2081, lung-derived mesothelioma cells. Such selective effects could be due to differences in these tumor cell lines (for example, HTB178 cells carry mutations in EGFR and p53, while CRL-2081 cells have amplified MYC activity). These preliminary results also suggest an attractive possibility that some of our small molecules, when given at a dose that can be tolerated by normal cells, could selectively kill certain tumor cells. We should note that many cancer drugs, such as doxorubicin, can damage DNA and are intrinsically cell-toxic.

### Perspective

Our high-throughput screen in human primary cells was designed to enrich for stress resistance-promoting small molecules that could potentially perturb pathways that affect lifespan. We characterized the hit compounds in several different cellular assays, and *in vivo*, in *C. elegans*. By doing these assays, we found several molecules, most notably the chalcone Gr-4D, that could be of potential interest for further translational studies.
